# Thromboelastometry as an Ancillary Tool for Evaluation of Coagulation Status after rFVIIa Therapy in a Pregnant Woman with Severe Hypoproconvertinemia—A Case Series and Review of the Literature

**DOI:** 10.3390/ijerph191710918

**Published:** 2022-09-01

**Authors:** Arkadiusz Krzyżanowski, Tomasz Gęca, Bożena Sokołowska, Maciej Kwiatek, Andrzej Miturski, Aleksandra Stupak, Piotr Terlecki, Piotr Paluszkiewicz, Anna Kwaśniewska

**Affiliations:** 1 Chair and Department of Obstetrics and Pathology of Pregnancy, Medical University of Lublin, 20-081 Lublin, Poland; 2 Chair and Department of Haematooncology and Bone Marrow Transplantation, Medical University of Lublin, 20-059 Lublin, Poland; 3 Chair and Department of Vascular Surgery and Angiology, Medical University of Lublin, 20-081 Lublin, Poland; 4 Department of General, Oncological and Metabolic Surgery, Institute of Haematology and Transfusion Medicine, 14 I. Gandhi Str., 02-776 Warsaw, Poland

**Keywords:** thromboelastometry, FVII deficiency, rFVIIa supplementation, venous thromboembolism, pregnancy

## Abstract

**Introduction:** Factor VII (FVII) deficiency is a rare hemorrhagic diathesis. In females, heavy menstrual and postpartum bleeding can appear as a consequence of its deficiency. Supplementation of the recombinant FVIIa is widely accepted. The supplementation effect in FVII-deficient subjects is difficult to predict, and severe hemorrhage has been described even when FVII levels after supplementation were within normal ranges. The aim of this report is to present the application of thromboelastometry to control the coagulation status in a patient with severe FVII deficiency during pregnancy and delivery, supplemented by rFVIIa per protocol complicated with life-threatening venous thromboembolism. **Methods:** Rotational thromboelastometry (ROTEM) was performed in 16 pregnant women: in one 28 year old primigravida at 35 weeks of pregnancy with congenital FVII deficiency after rFVIIa administration and 15 healthy women at 38 gestational weeks. The results were compared. **Results:** The thromboelastometry results showed significant shortening of the clotting time in the extrinsic and the intrinsic pathway in the hypoproconvertinemia patient after rFVIIa administration in relation to healthy pregnant women. A significant reduction in maximum lysis of the clot after FVII supplementation was observed. **Conclusions:** The thromboelastometry results showed a significant hypercoagulable state with hypoproconvertinemia. Thrombotic complications after delivery might be prevented by the reduction in rFVIIa guided by thromboelastometry. Thromboelastometry performed on a pregnant woman with factor VII deficiency during the supplementation of rFVIIa in peripartum time might be helpful in order to determine an individual, effective dosage regimen of rFVIIa to ensure full correction of clotting disorders without the tendency to develop thrombosis, but further studies are needed.

## 1. Introduction

Factor VII (FVII) deficiency is a rare hemorrhagic diathesis and is the third most frequent following von Willebrand disease and hemophilia. Hereditary FVII deficiency occurs in 1:500,000 of the overall population [1]. FVII exerts a key role in the activation of the clotting cascade in vivo [2]. The extrinsic coagulation pathway becomes activated due to tissue damage, forming a complex between active FVII (FVIIa) and the tissue factor (TF). The activation of factor X involves the activation of FVII. The circulating FVIIa constitutes approximately 1% of the total amount of FVII present in the bloodstream. Congenital factor VII deficiency is characterized by a decreased amount of the total FVII and a very low level of circulating FVIIa, practically undetectable in standard tests.The gene coding for FVII is located on the long arm of chromosome 13, in the vicinity of two genes, related to vitamin K-dependent proteins/blood coagulation factors, factor X (FX), and PROZ (protein Z, vitamin K-dependent plasma glycoprotein) [3,4]. The occurrence of FVII deficiency results from mutations taking place within the gene coding for FVII [4]. Over 250 causal mutations have been identified.

Recognizing FVII deficiency is performed on the basis of determining the isolated prothrombin time (PT), with the correct activated partial thromboplastin time (APTT) values. Raising the INR value (international normalized ratio) depends upon the plasma level of factor FVII coagulant (FVII:C). The final diagnosis is made on the basis of a decreased level of FVII in specialist tests.

Currently, certain types of FVII deficiency, e.g., those connected with Arg304Gln mutation (FVII Padua) are considered to cause little or no bleeding [5,6]. It seems that such patients should undergo substitution treatment in limited doses or none at all. Therefore, particular attention should be paid to recognize the exact type of coagulation impairment. Merely diagnosing FVII deficiency appears now to be insufficient [5,7]. Clotting disturbances should be characterized by means of at least three thromboplastins obtained from a rabbit brain, an ox brain, and a human placenta or a human recombinant thromboplastin [8,9]. The correct test finding, which uses thromboplastin obtained from an ox brain, is characteristic of Arg304Gln mutation (FVII Padua) and relatively uncommon mutation Arg304Trp (FVII Nagoya) [10].

The clinical picture of the hereditary FVII deficiency varies enormously, ranging from minor bleeding after surgeries to spontaneous, life-threatening hemorrhages [11]. Intense symptoms of hemorrhagic diathesis have been observed in homozygotes and compound heterozygotes of this feature, particularly in the case of FVII below 2% of the norm [2,12,13,14]. The disease is inherited autosomally and recessively; thus, it occurs in both men and women. In females, heavy menstrual and postpartum bleeding can appear as a consequence of FVII deficiency. Some researchers suggest that homozygotes are characterized by an FVII level below 10 IU/dL, whereas heterozygotes are characterized by an FVII level of 20–60 IU/dL [15].

In healthy females, during pregnancy, the concentrations of many clotting factors increase, sometimes even severalfold. In the case of FVII deficiency, this tendency pertains to heterozygotes and does not occur in homozygotes. The clinical symptoms of FVII deficiency vary tremendously and do not always correlate with its concentration. It is important to bear in mind that FVII deficiency does not protect against thromboembolic events [16,17,18], which may include both arterial and venous thrombosis with life threatening pulmonary embolism. Thromboembolic events appear in 3–4% of patients with FVII deficiency, not only in relation to a surgical operation or substitution treatment, but also spontaneously [16,17]. The group on hemostasis of the Polish Society of Hematologists and Transfusionists recommends maintaining the activity of FVII within the range of 5–10% in case of mild bleedings; however, when the patient is being prepared for a surgical operation, higher dosage regimens are necessary up to the level of 15–25% of the norm [19]. The medicine of choice is recombinant factor VIIa (rFVIIa) [20]. Prior to the introduction of the rFVIIa into treatment, the deficiency can be treated by preparations of fresh frozen plasma (FFP), concentrates of prothrombin factors (PCCs), and concentrates of FVII, derived from human plasma [21]. Using concentrates derived from human plasma involves the risk of transmitting viral infections. As a result of supplementing concentrates of prothrombin complex factors, other vitamin-K dependent factors are also supplemented, including II, IX, and X. Their levels dramatically increase in the patient’s body and lead to the risk of thrombotic complications, both arterial and venous ones [22].

rFVIIa was produced in order to treat patients suffering from hemophilia and FVIII inhibitor [7]. Its indications for use have been extended in Europe to patients suffering from FVII deficiency and Glanzmann’s thrombasthenia, refractory to platelet transfusions. The main argument in favor of the usefulness of using rFVIIa in patients with FVII deficiency is the fact that only the missing factor is provided. An additional point is the small volume of infusion and absence of risk in transmitting blood infections. FVII deficiency requires a substitution therapy which uses relatively moderate doses. The recommended dose, administered by intravenous bolus injection, is 15–30 μg/kg of body mass every 4–6 h [20], while, in hemophilia and FVIII inhibition, rFVIIa should be administered in the dose of 90 μg/kg of body mass, initially every 2–3 h. The discrepancy stems from the different pharmacokinetics of the medicine in such patients. In FVII deficiency, the drug clearance is faster, accompanied by an increased volume of distribution and smaller recovery. rFVIIa proved to be efficient not only in blocking but also in preventing bleeding. Regrettably, using rFVIIa in patients suffering from FVII deficiency is also linked with the occurrence of thrombotic complications. In order to make a realistic risk assessment, a group of researchers keeping an international registry of persons suffering from FVII deficiency (IRF7-International Registry on Factor VII Deficiency) and the rFVIIa manufacturer, Novo Nordisk company, designed the STER system (Seven Treatment Evaluation Registry) [16,20].

In the treatment of thrombosis in patients with FVII deficiency, by means of rFVIIa, low-molecular-weight heparin is most often exploited. Pregnant women with severe FVII deficiency who undergo a cesarean section, in most cases, receive rFVIIa as a preventive measure. Literature data devoted to this subject are also very scarce. It is difficult to precisely weigh the risks of thrombotic complications, paradoxically stemming from FVII deficiency, pregnancy, surgery, and lowering the concentration of this factor.

An attractive hypothesis might be a statement that a variety of causal mutations of FVII deficiency translates to a variety of clinical symptoms, including inclinations toward thrombosis [4]. It is considered that there is no strong link between FVII (FVII:C) activity and a tendency to bleed [1]. However, intensified bleeding/hemorrhage is usually observed in homozygotes and compound heterozygotes of this feature with FVII:C < 2% [2,13,14,15]. Raising the level of FVII:C to over 10–15% of the norm seems sufficient to ensure hemostasis [23].

In patients with known hereditary FVII deficiency, a supplementation of plasma-derived concentrate or rFVIIa is widely accepted. Clinical efficacy is usually observed when the level of the supplemented FVII is about 30% of normal. However, the supplementation effect in FVII-deficient subjects is difficult to predict, and severe hemorrhage has been described when FVII levels after supplementation were within normal ranges.

Thromboelastometry is an established whole-blood viscoelastic global hemostasis test. It investigates the interaction of coagulation factors, their inhibitors, anticoagulant drugs, and blood cells, specifically platelets, during clotting and subsequent fibrinolysis. Thromboelastometry consists of several specific tests including INTEM, HEPTEM, EXTEM, FIBTEM, and APTEM related to blood clotting induction methods. The use of viscoelastometric methods is currently an informative test for the identification of the hypercoagulable status and enhanced fibrinolysis during active bleeding and in patients with a high risk of perioperative blood loss [24,25]. In the available literature, there are not many publications on the use of these methods during pregnancy [24,26], and we did not find any data about the use of thromboelastometry in an FVII-deficient pregnant patient to control the coagulation status during supplementation of the deficient FVII.

The aim of this report is to present the application of thromboelastometry to control the coagulation status in a patient with severe FVII deficiency during pregnancy and delivery, supplemented by rFVIIa per protocol complicated with life-threatening venous thromboembolism.

## 2. Materials and Methods

Thromboelastometry was performed in 16 women: one pregnant patient with congenital FVII deficiency and 15 healthy pregnant women at 38 weeks of gestation without any coagulation disorders who were being hospitalized in the Department of Obstetrics and Pathology of Pregnancy, Medical University of Lublin. Each of the examined persons gave written informed consent to participate in the experiment. Research procedures were in line with ethical standards for human experimentation, consistent with the opinion of the Bioethics Committee of the Medical University of Lublin (No. KE-0254/347/2016) and with the Helsinki Declaration.

A 28 year old primigravida with congenital FVII deficiency was admitted to our department in the third trimester. The FVII deficiency was diagnosed in the patient at the age of 5 due to heavy bleeding after a tooth extraction. According to the medical history, the patient had heavy menstrual bleeding, treated with tranexamic acid and etamsylate. A few years before pregnancy, the patient underwent conservative treatment due to a bleeding episode in the lower part of the digestive tract, presumably after taking nonsteroidal, anti-inflammatory medication. The patient had never undergone any surgical operation. Before pregnancy, the activity of FVII remained at 0.52%. The pregnancy continued uninterrupted, and the patient remained under medical care of an experienced obstetrician and a hematologist. During the course of pregnancy, the patient had FVII activity monitored, which was equal to 0.65% at 7 weeks pregnancy, 0.78% at 15 weeks pregnancy, and 0.95% at 33/34 weeks pregnancy. At 35 weeks pregnancy, the patient was administered 2 mg of rFVIIa. FVII activity was equal to 3.04%. Due to the lack of standards in administering rFVIIa in patients with FVII deficiency, thromboelastometry was performed after drug administration.

Twenty milliliters of the whole blood was collected in 3.2% buffered sodium citrate. Two types of reagents were used for thromboelastometry (ROTEM, Pentapharm, Munich, Germany): EXTEM which involves tissue factor activation, INTEM which involves ellagic acid/phospholipid activation, and FIBTEM which is a combination of EXTEM and cytochalasine A. Cytochalasine A was used for the inactivation of platelets. The following ROTEM variables were estimated: clotting time (CT; s), clot formation (CFT; s), α-angle (°), maximum clot firmness (MCF; mm), and maximum lysis (ML). CT represented the onset of clotting, while CFT and α-angle both represented the initial rate of fibrin polymerization. MCF was a measure of the maximal viscoelastic strength of clot. ML was estimated in the 60th minute of the test for the diagnosis of premature lysis of hyperfibrinolysis.

**Figure 1:**
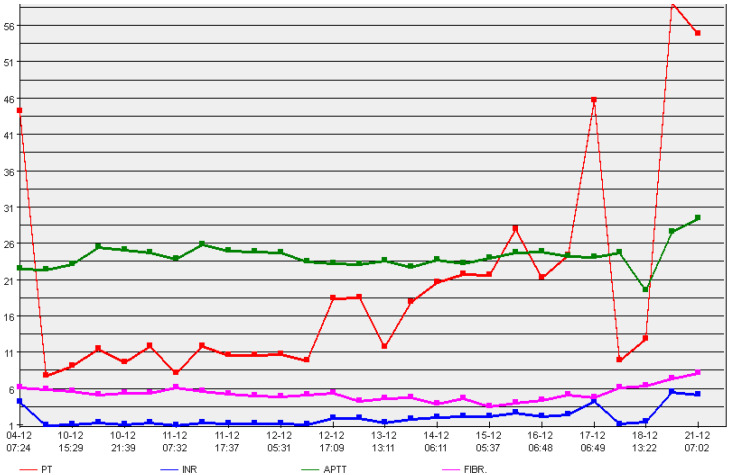
Coagulogram. Prothrombin time—PT, international normalized ratio—INR, activated partial thromboplastin time—APTT, fibrinogen—Fibr.

On the basis of the obtained findings of thromboelastometry, after intravenously administering 2 mg of rFVIIa to the described patient and comparing the obtained results with thromboelastometry data in healthy pregnant women, a hematologist, an obstetrician, and a hemorrhage consultant jointly determined the dosage and therapeutic management. The thromboelastometry results showed a slight hypercoagulable state after rFVIIa administration. However, no protocols indicated the necessity for FVII dose correction based on elastometric findings. At the time of the patient’s hospitalization (from 37 weeks 1 day of gestation until the 11th day of puerperium), a coagulogram was regularly monitored (Figure 1).

At 38+0 weeks of pregnancy, under general endotracheal anesthesia, a cesarean section was performed because of symptoms of intrauterine hypoxia. A full-term live-born female newborn was delivered, 2840 g in weight. The dosage of rFVIIa, tranexamic acid, and nadroparin is presented in Table 1.

On the sixth day, the patient complained of redness and pain of the right calf in the lower part of the right leg. The conducted USG Doppler revealed thrombosis of venous sinuses in the calf gastrocnemius muscle. Compression therapy was recommended, i.e., compression stockings grade 2, as well as movement activation. The administration of tranexamic acid was abandoned. However, nadroparin in the dosage of 2 × 0.3 mL was introduced. During the next day, a follow-up examination revealed progression of changes—the presence of thrombosis was diagnosed, which spread over the confluence of tibial and fibular veins and popliteal vein, with a free-floating thrombus head. Due to ultrasonographic symptoms of free-floating thrombus, it was decided to immobilize the patient for a period of 2 days. The treatment on days 7 and 8 is illustrated in Table 1. Despite the conducted treatment, on day 9, symptoms of moderate dyspnea at rest, cough, and chest pains occurred. The patient had spiral CT angiography of the pulmonary arteries performed urgently, in the layers of 0.625 mm, after an intravenous administration of contrast, which allowed diagnosing pulmonary embolism within segments 9 and 10 of the right lung and single subsegmental ones within segments 3 and 6 of the right lung. Moreover, parenchymal density and atelectatic lesions within segments 9 and 10 of the right lung were detected. In addition, a minor strain of atelectasis, peripherally within segments 9 and 10 of the left lung, was noticed. The patient was transferred to the Clinic of Vascular Surgery and Angiology. The administration of rFVIIa was abandoned, and nadroparin was further administered in the dosage of 2 × 0.6 mL until the day of leaving the hospital (13 day after delivery).

An additional examination revealed lowered parameters in a blood gas analysis—pCO_2_ 30.9 mmHg, pO_2_ 73.50 mmHg, sO_2_ 95.4%, pH 7.448, D-dimers 5800 UE/mL, and a slightly lowered level of FVII 42.3%; rFVIIa did not need any further infusions.

The patient was discharged home in good condition with a complete regression of clinical symptoms of pulmonary embolism. She was administered nadroparin, in the dosage of 2 × 0.6 mL, until day 17 after delivery, and then 1 × 0.6 mL for the next 6 weeks.

The patient remained under constant care of a specialist, a vascular surgeon; within a 7 month observation, full recanalization of the popliteal vein and the confluence of tibial and fibular veins were observed. At present, in secondary antithrombotic prevention, the patient is using compression stockings grade 2. An obstetrical check-up at 6 and 12 weeks after delivery did not show any abnormalities. The hematological examinations of the neonate in the sixth and 12th months of her life did not detect any hypoproconvertinemia.

## 3. Results

The thromboelastometry results showed a significant shortening of the clotting time in EXTEM and INTEM probes in the hypoproconvertinemia patient after rFVIIa administration in relation to healthy pregnant women (Table 2).

The ROTEM parameters represent 15 healthy pregnant women and one case of FVII severe deficiency in a pregnant woman. The tests were performed at 38 weeks of pregnancy. The thromboelastometry in the FVII-deficient woman was completed directly after rFVIIa infusion before delivery. ΔD—the difference between FVII deficiency and median of healthy, calculated for each row in the table as follows: (FVII deficiency − median of healthy) × 100%/median of healthy. The minus value indicates shortening of the recorded value in the studied case with FVII deficiency. CT—clotting time; CFT—clotting formation time; alpha—the angle representing the amplitude increase during clot formation in the sample; A10—amplitude in 10th minute of test; A20—amplitude in 20th minute of test; MCF—maximum clot firmness; ML maximum lysis observed in 60th minute of test.Moreover, we observed a significant reduction in maximum lysis of the clot after FVII supplementation.

## 4. Discussion

Medical care during pregnancy of women with hemorrhagic diathesis is an extremely difficult task. It requires cooperation of numerous specialists, particularly of an obstetrician and a hematologist, access to a specialist laboratory, and possibilities of using modern substitution treatment. Hemorrhagic diathesis is quite uncommon; therefore, there are no readymade standards of therapeutic management. In the presented case, at 35 weeks of pregnancy, fibroelastometry was performed. The conducted analysis of thromboelastometry, after the supplementation of rFVIIa, in the presented patient with hypoproconvertinemia indicated significant shortening of the clotting time and initial formation of a thrombus (CT) in the global blood test. rFVIIa protected the thrombus against the fibrinolytic system, lowering the indicator of clot lysis in the global test (ML). This effect was not so obvious when platelets became eliminated from the examination (FIBTEM test). Thus, it leads to a conclusion that the plasma coagulation system after rFVIIa infusion, in patients with hypoproconvertinemia, shows an intensified activity, in both extrinsic and intrinsic pathways, which may be interpreted as plasma hypercoagulation and a tendency to thrombosis. It is interesting to note the protective role of blood platelets, which stop excessive clot lysis, observed in EXTEM and INTEM in the ML test. However, this was unnoticed in FIBTEM test, where it remained minimal.

Tranaxemic acid (TXA) is effective for reducing blood loss and the need for transfusions during surgeries. Clotting time during the postoperative period is shorter and clot strength is greater in patients treated with TXA. It can also prevent the increase in D-dimer levels without affecting systemic hemostasis [27]. In our patient, we observed that clotting time was shorter in comparison to healthy pregnant not receiving TXA. However, it is difficult to say definitely whether this was due to TXA or rFVIIa.

The discussed patient had homozygous FVII deficiency, with the factor level below 1%, which did not rise significantly in the other half of the pregnancy or before delivery. The major complication was the occurrence of thromboembolism. It needs to be stressed that pregnancy and a cesarean section carry the risk of thrombotic complications, and FVII deficiency does not prevent thrombotic complications. Girolami et al. in 2010 analyzed cases of occurring thrombotic complications in patients with FVII deficiency [18]. They discovered that, in most patients, thrombosis coincides with risk factors, such as injury, prolonged immobility, substitution treatment mostly with a concentrate of prothrombin complex (PCC), lipid disturbances, smoking, and diabetes.

The patient in question, despite heavy deficiency of FVII, had the symptoms of hemorrhagic diathesis slightly intensified. However, an additional prothrombotic factor was poor activity of the patient after a cesarean section, as well as using rFVIIa. It needs to be stressed that, although the most advantageous ending of the pregnancy would have been a natural delivery, it was necessary to perform a cesarean section due to the symptoms of intrauterine hypoxia. The recommended dose of rFVIIa in connection with a surgical operation is 8–40 U/kg body mass, every 4–6 h (the half-life of FVII is equal to 3–4 h) or 30–40 U/kg body mass every 12 h until the wound heals [19]. A scrutiny of scientific data revealed that, so far, 14 patients with FVII deficiency have been described worldwide, objectively diagnosed with pulmonary embolism [16,17,28]. In 12 cases, the patients were homozygous, while they were compound heterozygous in two cases. In five cases, the patients were treated with low-molecular-weight heparin or unfractionated heparin or coumarin derivatives, while undergoing substitution treatment.

On the basis of available case studies of venous thromboembolism in patients with inherited FVII deficiency, treated by means of rFVIIa, it is impossible to draw a conclusion whether using this preparation causes a risk increase in the occurrence of a thrombotic episode.

In one systematic review of the management of pregnancy in FVII-deficient women, no difference in post-partum hemorrhage was seen in deliveries with and without prophylaxis [29]. Therefore, the authors recommend that rFVIIa should be made available in the case of hemorrhage or surgical intervention, but not as mandatory prophylaxis. In the research based on a series of five patients, Lee et al. suggested that the management of delivery for women with FVII deficiency should be addressed on a case-by-case basis and at centers with expertise in rare bleeding disorders [30]. In two other recently published articles, peripartum prophylaxis was strongly recommended [31,32].

## 5. Conclusions

In our particular case, the thromboelastometry results showed a significant hypercoagulable state in the patient with hypoproconvertinemia treated with recombinant activated factor VII. The standard clinical course performed per protocol with the use of a widely recommended dose of rFVIIa was complicated with life-threatening venous thromboembolism. Patients at risk of thrombosis should be routinely given a prophylactic dose of low-molecular-weight heparin. However, in the case of the described patient, such prophylaxis was not applied due to the high risk of perinatal hemorrhage. Low-molecular-weight heparin was introduced only when symptoms of thrombosis appeared. We assumed that thrombotic complications after delivery might have been prevented by the reduction in rFVIIa guided by thromboelastometry. Thromboelastometry performed on a pregnant woman peripartum with factor VII deficiency during the supplementation of rFVIIa might be an ancillary tool in order to determine the individual, effective dosage regimen of rFVIIa to ensure full correction of clotting disorders without a tendency to develop thrombosis. We are aware of the limited number of patients involved in the study, highlighting the need for further studies.

## Figures and Tables

**Table 1:** Dosage of recombinant activated factor VII, tranexamic acid, and nadroparin after delivery on days 1–14.

	Day									
**Treatment**	0	1	2	3	4	5	6	7	8	9–14
**Recombinant** **activated factor VII**	2 mg every 8 h	2 mg every 12 h	2 mg every 12 h	2 mg every 12 h	2 mg every 12 h	1 mg every 12 h	1 mg every 12 h	1 mg every 24 h	1 mg every 24 h	1 mg every 24 h
**Tranexamic acid**	1 g every 8 h	1 g every 8 h	1 g every 8 h	1 g every 12 h	1 g every 12 h	1 g every 24 h	-	-	-	-
**Nadroparin** **0.3 mL = 2850 U**	-	-	-	-	-	-	-	0.3 mL every 12 h	0.3 mL every 12 h	0.6 mL every 24 h

**Table 2:** Thromboelastometry results.

Healthy																	FVII Deficiency	ΔD
	1	2	3	4	5	6	7	8	9	10	11	12	13	14	15	Median (Min–Max)	One Case	
**EXTEM**																		
CT	65	65	65	65	65	50	53	59	57	57	50	67	63	60	55	60 (50–67)	35	−42
CFT	55	53	59	71	77	63	107	52	73	60	70	69	67	64	65	65 (52–107)	80	23
alfa	79	79	78	76	77	80	68	79	77	78	76	80	78	76	77	78 (68–80)	78	0
A10	67	67	65	60	56	60	52	76	57	62	62	55	59	60	58	60 (52–76)	54	−10
A20	71	72	70	64	62	67	59	71	62	63	69	63	66	64	64	66 (59–72)	62	−6
MCF	71	73	70	64	64	68	60	71	64	60	70	60	67	65	66	67 (60–73)	65	−3
MI.	16	6	7	16	12	2	12	0	9	8	14	8	12	10	13	10 (0–16)	3	−70
**INTEM**																		
CT	167	149	130	136	154	160	472	161	328	192	118	145	118	182	404	160 (118–472)	95	−41
CFT	40	57	48	62	66	72	117	49	117	60	67	58	64	64	103	64 (40–117)	67	5
alfa	80	78	81	78	82	78	67	80	67	78	76	79	79	80	69	78 (67–82)	79	1
A10	65	62	64	59	55	55	48	64	51	62	60	59	58	64	52	59 (48–65)	56	−5
A20	70	67	68	62	61	61	55	69	59	68	65	63	64	68	59	64 (55–70)	63	−2
MCF	70	67	68	62	62	62	56	70	61	69	65	64	65	70	61	65 (56–70)	64	−2
MI.	9	9	8	16	15	4	15	1	7	6	13	10	12	7	13	9 (1–16)	4	−56
**FISTEM**																		
CT	58	63	55	59	54	45	47	50	49	44	40	60	64	45	44	50 (40–64)	32	−36
CFT	118	412	340	909	649	376		69	536	116	359	407	142	120	990	368 (69–990)	209	−43
alfa	78	79	78	74	77	80	74	83	74	74	77	79	80	75	75	77 (74–83)	80	4
A10	25	21	22	19	20	21	15	24	21	34	21	23	25	32	19	21 (15–34)	23	10
A20	26	22	23	21	22	22	17	25	21	38	22	24	27	34	20	22 (17–38)	25	14
MCF	26	23	24	21	22	22	17	26	22	39	22	26	28	35	21	23 (17–39)	26	13
MI.	0	0	1	2	0	0	0	13	2	10	0	0	0	9	0	0 (0–13)	0	0

## Data Availability

The data presented in this study are available on request from the corresponding author.

## Abbreviations

aFVII	Active factor VII
APTT	Activated partial thromboplastin time
CT	Clotting time
CFT	Clot formation time
EXTEM	Tissue factor activation
FVII	Factor VII
FX	Factor X
FIBTEM	Combination of EXTEM and cytochalasine A
INR	International normalized ratio
INTEM	Ellagic acid/phospholipid activation
MCF	Maximum clot firmness
ML	Maximum lysis
PCC	Prothrombin complex
PROZ	Protein Z, vitamin K-dependent plasma glycoprotein
PT	Prothrombin time
rFVIIa	Recombinant factor VIIa
ROTEM	Rotational thromboelastometry
TF	Tissue factor
TXA	Tranaxemic acid

## References

[1] Mariani G., Dolce A., Lee C., Berntorp E., Hoots K. (2005). Congenital factor VII deficiency. Textbook of Hemophilia.

[2] Bauer K.A. (1996). Treatment of factor VII deficiency with recombinant factor VIIa. Haemostasis.

[3] O’Hara P.J., Grant F.J., Haldeman B.A., Gray C.L., Insley M.Y., Hagen F.S., Murray M.J. (1987). Nucleotide sequence of the gene coding for human factor VII, a vitamin K-dependent protein participating in blood coagulation. Proc. Natl. Acad. Sci. USA.

[4] Mariani G., Bernardi F. (2009). Factor VII Deficiency. Semin. Thromb. Hemost..

[5] Girolami A., Fabris F., Zanon R.D.B., Ghiotto G., Burul A. (1978). Factor VII Padua: A congenital coagulation disorder due to an abnormal factor VII with a peculiar activation pattern. J. Lab. Clin. Med..

[6] Kirkel D., Lin T.-W., Fu S.W., Dlott J.S., Sahud M.A., McCaffrey T., Rickles F.R. (2010). Asymptomatic factor VII deficiency: Gene analysis and structure-function relationships. Blood Coagul. Fibrinolysis.

[7] Perry D.J. (2002). Factor VII deficiency. Br. J. Haematol..

[8] Triplett D.A., Brandt J.T., Batard M.A., Dixon J.L., Fair D.S. (1985). Hereditary factor VII deficiency: Heterogeneity defined by combined functional and immunochemical analysis. Blood.

[9] Girolami A., Bonamigo E., Vettore S. (2010). The lack of ties between north-eastern Italy and African-Americans suggest a multifounder effect for FVII Padua (Arg304Gln) disorder. Blood Coagul. Fibrinolysis.

[10] Matsushita T., Kojima T., Emi N., Takahashi I., Saito H. (1994). Impaired human tissue factor-mediated activity in blood clotting factor VII Nagoya (Arg304–>Trp). Evidence that a region in the catalytic domain of factor VII is important for the association with tissue factor. J. Biol. Chem..

[11] Kulkarni A.A., Lee C.A., Kadir R.A. (2006). Pregnancy in women congenital factor VII deficiency. Haemophilia.

[12] McVey J.H., Boswell E., Mumford A.D., Kemball-Cook G., Tuddenham E.G. (2001). Factor VII deficiency and the FVII mutation database. Hum. Mutat..

[13] Tcheng W.Y., Donkin J., Konzal S., Wong W.-Y. (2004). Recombinant factor VIIa prophylaxis in a patient with severe congenital factor VII deficiency. Haemophilia.

[14] Mariani G., Herrmann F.H., Dolce A., Batorova A., Etro D., Peyvandi F., Wulff K., Schved J.F., Auerswald G., Ingerslev J. (2005). Clinical phenotypes and FVII genotypes congenital factor VII deficiency. Thromb. Haemost..

[15] Kolucki F.R., Morris G.J., Thomas L.C., Scialla S. (2011). Factor VII deficiency in pregnancy and delivery: A case report. Haemophilia.

[16] Mariani G., Herrmann F.H., Schulman S., Batorova A., Wulff K., Etro D., Dolce A., Auerswald G., Astermark J., Schved J.F. (2003). Thrombosis in inherited factor VII deficiency. J. Thromb. Haemost..

[17] Marty S., Barro C., Chatelain B., Fimbel B., Tribout B., Reynaud J., Schved J.F., Giansily-Blaizot M. (2008). The paradoxical association between inherited factor VII deficiency and venous thrombosis. Haemophilia.

[18] Girolami A., Tezza F., Scandellari R., Vettore S. (2010). Associated prothrombotic conditions are probably responsible for the occurrence of thrombosis in almost all patients with congenital FVII deficiency. Critical review of the literature. J. Thromb. Thrombolysis.

[19] Zawilska K., Chojnowski K., Klukowska A., Łętowska M., Mital A., Musiał J., Podolak-Dawidziak M., Undas A., Windyga J., Zdziarska J. (2011). Polskie zalecenia postępowania w rzadkich niedoborach osoczowych czynników krzepnięcia. Hematologia.

[20] Napolitano M., Giansily-Blaizot M., Dolce A., Schved J.F., Auerswald G., Ingerslev J., Bjerre J., Altisent C., Charoenkwan P., Michaels L. (2010). Prophylaxis congenital factor VII deficiency, indication, efficacy and safety: Results of the STER. Blood.

[21] Ingerslev J., Kristensen H.L. (1998). Clinical picture and treatment strategies in factor VII deficiency. Haemophilia.

[22] Aledort L.M. (2004). Comparative thrombotic event incidence after infusion of recombinant factor VIIa versus factor VIII inhibitor bypass activity. J. Thromb. Haemost..

[23] Ingerslev J., Knudsen L., Hvid I., Tange M.R., Fredberg U., Sneppen O. (1997). Use of recombinant factor VIIa in surgery in factor-VII-deficient patients. Haemophilia.

[24] Nakayama Y., Nakajima Y., Tanaka K.A., Sessler D.I., Maeda S., Iida J., Ogawa S., Mizobe T. (2015). Thromboelastometry-guided intraoperative haemostatic management reduces bleeding and red cell transfusion after paediatric cardiac surgery. Br. J. Anaesth..

[25] Naik B.I., Pajewski T.N., Bogdonoff D.I., Zuo Z., Clark P., Terkawi A.S., Durieux M.E., Shaffrey C.I., Nemergut E.C. (2015). Rotational thromboelastometry-guided blood product management in major spine surgery. J. Neurosurg. Spine.

[26] Carrabin N., Benchaib M., Fontaine O., Levrat A., Massignon D., Touzet S., Rudigoz R.-C., Berland M., Huissoud C. (2009). Coagulation assessment by rotation thrombelastometry in normal pregnancy. Thromb. Haemost..

[27] Kim E.J., Kim Y.O., Shim K.W., Ko B.W., Lee J.W., Koo B.-N. (2018). Effects of Tranexamic Acid Based on its Population Pharmacokinetics in Pediatric Patients Undergoing Distraction Osteogenesis for Craniosynostosis: Rotational Thromboelastometry (ROTEM^TM^) Analysis. Int. J. Med Sci..

[28] Girolami A., Berti de Marinis G., Vettore S., Girolami B. (2013). Congenital FVII Deficiency and Pulmonary Embolism: A Critical Appraisal of All Reported Cases. Clin. Appl. Thromb. /Hemostasis.

[29] Baumann Kreuziger L.M., Morton C.T., Reding M.T. (2013). Is prophylaxis required for delivery in women with factorVIIdeficiency?. Haemophilia.

[30] Lee E., Burey L., Abramovitz S., DeSancho M.T. (2020). Management of pregnancy in women with factorVIIdeficiency: A case series. Haemophilia.

[31] Yang Y., Zeng Y.C., Rumende P., Wang C.G., Chen Y. (2021). Diagnosis and treatment discussion of congenital factor VII deficiency in pregnancy: A case report. World J. Clin. Cases.

[32] Loddo A., Cornacchia S., Cane F.L., Barcellona D., Marongiu F., Melis G.B., Angioni S., Paoletti A.M., Neri M. (2019). Prophylaxis of peripartum haemorrhage using recombinant factor VIIa (rfVIIa) in pregnant women with congenital factor VII deficiency: A case report and literature review. Eur. J. Obstet. Gynecol. Reprod. Biol..

